# Cutaneous Wound Healing Facilitated by Postbiotic Extract Through Antimicrobial Action and Extracellular Matrix Regulation

**DOI:** 10.3390/ijms262110556

**Published:** 2025-10-30

**Authors:** Wanning Zhang, Wenhao Yu, Xixian Li, Yang Yu, Jingwen Feng, Yinghang Xu, Muxin Zhao, Yan Jin

**Affiliations:** 1 Division of Energy Research Resources, Dalian Institute of Chemical Physics, Chinese Academy of Sciences, Dalian 116023, China; zhangwanning@dicp.ac.cn (W.Z.); yuwenhao@dicp.ac.cn (W.Y.); yuyang1809@dicp.ac.cn (Y.Y.); fengjingwen@dicp.ac.cn (J.F.); 2 Dalian Medical University, Dalian 116044, China; lianjinyuansu@foxmail.com (X.L.); nightfiery010@outlook.com (Y.X.)

**Keywords:** wound healing, postbiotic extract, antimicrobial properties, anti-inflammatory effects, keratinocyte proliferation

## Abstract

Wound healing is a multifaceted biological process that involves a series of cellular interactions and immune responses to restore tissue integrity following injury. Postbiotics, non-viable microbial products, have garnered increasing attention for their potential therapeutic applications in wound healing. This study investigated the efficacy of a postbiotic extract derived from *Lactobacillus bulgaricus* (*L. bulgaricus*) fermentation in promoting wound healing. The extract was prepared by controlled fermentation, followed by inactivation and purification. In vitro, we assessed antimicrobial properties against *Escherichia coli* (*E. coli*), *Staphylococcus aureus* (*S. aureus*), and *Pseudomonas aeruginosa* (*P. aeruginosa*) and performed peptidomic analysis to identify antimicrobial peptides. Effects on HaCaT proliferation, immune modulation, and collagen synthesis were evaluated. In vivo, a full-thickness *S. aureus*–infected wound model in C57BL/6 mice was used to assess wound closure and collagen deposition. Together, the in vitro and in vivo findings demonstrated antimicrobial, immunomodulatory, and regenerative activities, supporting composite postbiotics as a multifunctional wound-care approach.

## 1. Introduction

Wound healing is a complex and tightly regulated biological process that restores the integrity of damaged tissue [1,2]. It proceeds through four distinct but overlapping phases: hemostasis, inflammation, proliferation, and remodeling, each contributing to the restoration of tissue structure and function [1,3,4]. This process is orchestrated by multiple cells types, including keratinocytes, fibroblasts, and immune cells, as well as a wide range of biochemical signals and growth factors [5]. Hemostasis is initiated by platelet plug formation and the release of clotting factors to prevent blood loss. The subsequent inflammatory phase is characterized by the recruitment of neutrophils and macrophages, which remove cellular debris and pathogens [6,7,8] and release cytokines that trigger the repair process [9]. The proliferative phase is marked by granulation tissue formation, re-epithelialization, and extracellular matrix (ECM) deposition. Finally, during the remodeling phase, the ECM is reorganized, leading to scar tissue formation and the gradual restoration of tissue functionality [3,8]. Despite extensive research into the cellular and molecular mechanisms of wound healing, traditional treatment strategies remain limited, particularly for chronic wounds such as diabetic, venous and pressure ulcers [10,11]. The persistence of such wounds increases the risk of infection, chronic inflammation, and scarring, underscoring the need for more effective therapeutic strategies.

Lactic acid bacteria (LAB) produce bioactive substances, such as short-chain fatty acids and peptides, through their metabolic processes, which confer various health benefits [12,13]. Traditionally, probiotics have been developed as foods or dietary supplements to promote gut health [13]. However, recent studies suggest that LAB may also contribute to wound care by facilitating wound bed cleansing, reducing inflammation, and accelerating epithelial cell migration and proliferation [14,15,16]. Despite these advantages of LAB fermentation, live LAB are generally not preferred for wound care, especially in cases of compromised skin barriers, due to safety concerns [14,17,18,19]. Consequently, there remains a need for therapeutic strategies that capture the benefits of LAB without the risks associated with viable microorganisms. Postbiotics have emerged as a promising solution. These preparations contain non-viable microbial cells and their metabolites, retaining bioactivity while eliminating the risk of infection. Moreover, compared with single isolated components, composite postbiotic extracts include a broader spectrum of functional molecules, including peptides, organic acids, and exopolysaccharides, which may act synergistically to regulate inflammation, stimulate keratinocyte proliferation, and modulate ECM remodeling [20]. Several studies have shown that postbiotics can significantly improve the rate and quality of wound healing by enhancing the epithelial barrier, modulating immune responses, and promoting cell proliferation [21,22,23]. Therefore, this study aimed to evaluate the effects of a postbiotic extract derived from *Lactobacillus bulgaricus* (*L. bulgaricus*) on wound healing, particularly in the context of involving bacterial-induced inflammation or immune-mediated responses, and to assess its potential to accelerate wound repair.

## 2. Results

### 2.1. Antimicrobial Activity of the Postbiotic Extract In Vitro

**Figure 1:**
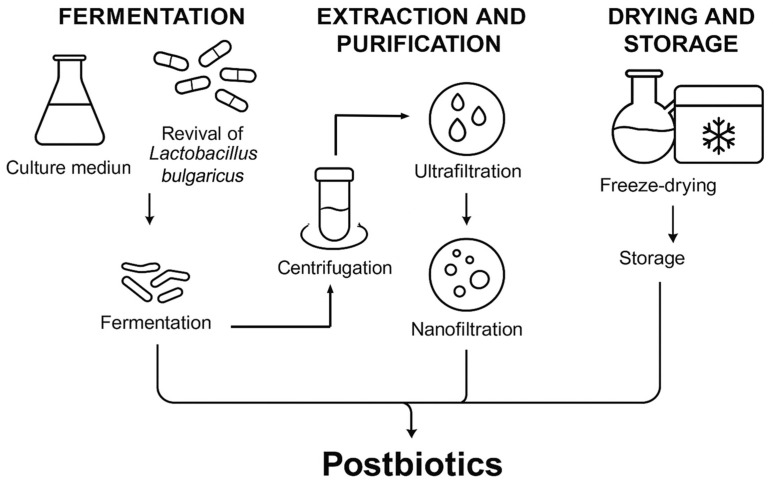
Schematic representation of the postbiotic extract preparation process. The process involved fermentation of *L. bulgaricus* in reconstituted skim milk, followed by centrifugation, ultrafiltration, nanofiltration, freeze-drying, and low-temperature storage.

As shown in Figure 1, the postbiotic extract was prepared and initially validated for biological activity. The minimum inhibitory concentrations (MICs) of the extract were determined against three common wound-associated pathogens. The postbiotic extract inhibited both Gram-positive and Gram-negative bacteria. It exhibited strong inhibitory activity against *Escherichia coli* (*E. coli*) (MIC 6.4 mg/mL). In contrast, its effects on *Staphylococcus aureus* (*S. aureus*) and *Pseudomonas aeruginosa* (*P. aeruginosa*) were less pronounced, requiring higher concentrations to achieve growth inhibition (Table 1). These differences may have reflected species-specific physiological traits and intrinsic resistance mechanisms. *S. aureus* is well known for its significant antibiotic resistance and adaptability, whereas *P. aeruginosa* is intrinsically resistant to multiple antibiotics [24,25].

### 2.2. Peptide Identification and Compositional Analysis in the Postbiotic Extract

To reveal the composition of the postbiotic extract, we analyzed its protein and total soluble sugar contents (Table 2). The protein content was 9.85 ± 0.49 g/100g and the total soluble sugar content was 50.73 ± 1.29 g/100g in the postbiotic extract, with lactose accounting for 46.88 ± 1.85 g/100g. The raw skim milk powder used as the fermentation substrate contained 35.74 ± 0.29 g/100 g of protein, 38.61 ± 2.13 g/100 g of total soluble sugars, and 32.21 ± 0.55 g/100 g of lactose. These data indicated that the majority of the soluble sugars in the postbiotic extract originated from the substrate. Compared to the substrate, the postbiotic extract contained more total soluble sugars and less protein. During fermentation, lactose served as the main supply source for microorganism growth. Although some lactose was metabolized, it remained the predominant sugar in the postbiotic extract. In addition, polysaccharides produced by LAB during fermentation contributed to the soluble sugar fraction. These polysaccharides may have bioactive properties, but their functional roles were beyond the scope of this study [26].

Considering that the antimicrobial peptides (AMPs) have attracted increasing attention, fermentation was one of the methods to produce AMPs [27]. Therefore, the peptides and AMPs in the postbiotic extract were analyzed. Using LC-MS/MS, a total of 888 peptides were identified. More than 43% of these peptides were derived from *β*-casein, while peptides from κ-casein and *α*S1-casein together accounted for approximately 18% of the total. Using the MBPDB database, we identified eleven AMPs among the peptides, representing 4.69% of the total abundance [28,29]. The peptide YQEPVLGPVRGPFPIIV exhibited the highest relative abundance at 3.47% and was considered the primary potential antimicrobial component, followed by DVENLHLPLPL at 0.51% (Table 3). These peptides possessed sequence features enabling membrane binding and disruption, thereby exerting bactericidal effects against both Gram-positive and Gram-negative bacteria. They were likely key contributors to the antimicrobial activity of the postbiotic extract.

### 2.3. Anti-Inflammatory Activity of the Postbiotic Extract In Vitro

**Figure 2:**
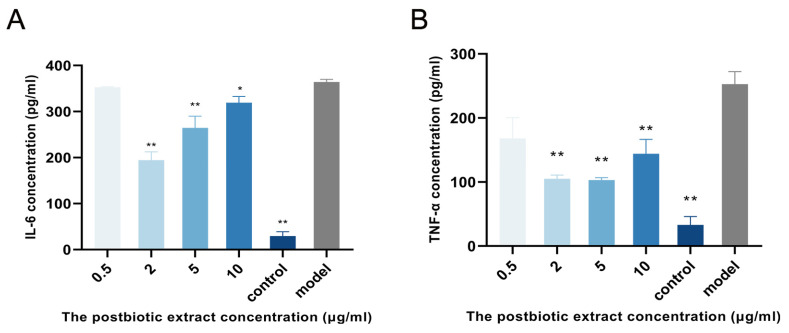
Effect of the postbiotic extract on inflammatory cytokine secretion in LPS-stimulated RAW264.7 cells. (**A**) Inhibition of IL-6 secretion by the postbiotic extract. RAW264.7 cells were stimulated with LPS and subsequently treated with the postbiotic extract (0.5, 2, 5, 10 µg/mL) or PBS (model group). The control group was not stimulated with LPS. * *p* < 0.05, ** *p* < 0.01 vs. model. (**B**) Inhibition of TNF-*α* secretion by the postbiotic extract. TNF-*α* levels were assessed using the same procedure as in (**A**). * *p* < 0.05, ** *p* < 0.01 vs. model.

Inflammation is one of the key phases in the wound-healing process, and agents with anti-inflammatory properties can accelerate tissue repair. Therefore, the anti-inflammation activity of the postbiotic extract was investigated. To model inflammation, lipopolysaccharide (LPS) was used to stimulate RAW264.7 macrophages, mimicking the inflammatory milieu typically encountered in bacterial infections. As shown in Figure 2, IL-6 and TNF-*α* levels were significantly higher in the LPS-stimulated group than in the untreated control group, confirming successful model establishment. IL-6 secretion decreased by 46% at 2 μg/mL and by 27% at 5 μg/mL. Similarly, TNF-*α* levels decreased to 26%, 40%, 41%, and 46% of the model group at 0.5, 2, 5, and 10 μg/mL, respectively. Notably, the 2 μg/mL treatment produced the most pronounced simultaneous reduction in both IL-6 and TNF-*α*, suggesting that this concentration may have been the most effective for modulating inflammatory responses in vitro.

### 2.4. Skin Cell Proliferative Activity of the Postbiotic Extract In Vitro

**Figure 3:**
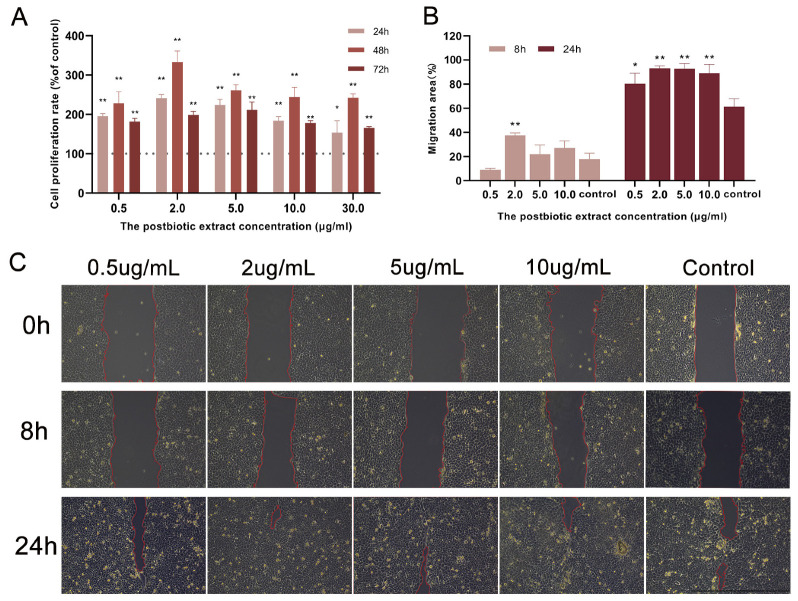
Effect of the postbiotic extract on HaCaT cell proliferation. (**A**) Proliferative effect of the postbiotic extract on HaCaT cells. Cells were treated with the postbiotic extract (0.5, 2, 5, 10, 30 µg/mL) or PBS (control, set as 100%). * *p* < 0.05, ** *p* < 0.01 vs. control. (**B**,**C**) Representative images and quantification of the scratch wound-healing assay in HaCaT cells treated with the postbiotic extract (0.5, 2, 5, and 10 µg/mL) or PBS (control) for 8 and 24 h. * *p* < 0.05, ** *p* < 0.01 vs. control (scale bar = 1000 μm).

Considering that cell proliferation is a critical determinant of wound healing rate and quality, the effects of the postbiotic extract on HaCaT cells in this process were explored. In a HaCaT proliferation assay, treatment with the postbiotic extract (0.5–10 µg/mL) significantly increased cell viability (Figure 3A). After 24 h, 2 µg/mL of the extract induced a 93% increase in cell proliferation. The proliferative response peaked at 48 h, with proliferation indices of 1.73-, 2.54-, 1.99-, 1.86-, and 1.84-fold relative to the control at 0.5, 2, 5, 10 and 30 µg/mL, respectively. At 72 h, significant proliferation was observed at 2 and 5 µg/mL, corresponding to an approximate 50% increase.

To further assess the wound-healing potential of the postbiotic extract, a scratch assay was conducted. At 8 h, the wound closure rate in the control group was 17.8%, whereas treatment with 2 µg/mL of the extract significantly increased closure to 37.42%. After 24 h, the control group had a closure rate of 61.39% in the control group and 93.19%, 92.86%, and 88.98% in cells treated with 2, 5, and 10 µg/mL of the extract, respectively (Figure 3B,C). These findings indicate that the postbiotic extract significantly promoted keratinocyte migration, with the most pronounced effects observed at 2–5 µg/mL.

### 2.5. Effects of the Postbiotic Extract on Type I Collagen (I-COL) and Matrix Metalloproteinases (MMPs) Expression In Vitro

**Figure 4:**
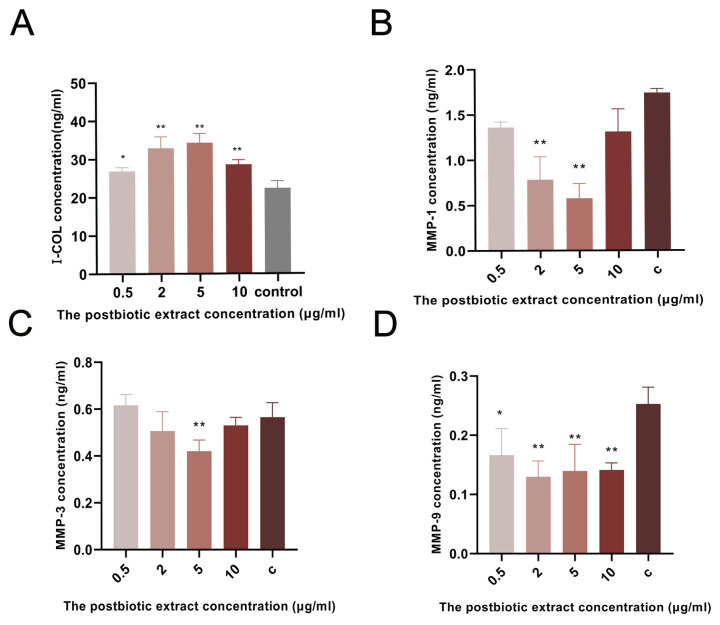
Effect of the postbiotic extract on I-COL and MMP secretion. (**A**) Secretion of I-COL by HaCaT cells treated with the postbiotic extract. Cells were treated with the postbiotic extract (0.5, 2, 5, 10 µg/mL) or PBS (control). * *p* < 0.05, ** *p* < 0.01 vs. control. (**B**–**D**) Secretion of MMPs (MMP-1, MMP-3, MMP-9) by HaCaT cells treated with the postbiotic extract. Cells were treated with the postbiotic extract (0.5, 2, 5, 10 µg/mL) or PBS (control). * *p* < 0.05, ** *p* < 0.01 vs. control.

I-COL plays an important role in wound healing by forming new collagen fibers and promoting tissue repair and skin regeneration. The postbiotic extract markedly influenced ECM remodeling in HaCaT cells. Compared with the control group, I-COL levels increased by 20.3%, 47.3%, 52.4%, and 27.9% at 0.5, 2, 5, and 10 µg/mL, respectively (Figure 4A). In parallel, MMP secretion was substantially reduced. MMP-1 levels decreased by 21.8%, 55.2%, and 66.8% at 0.5, 2, and 5 µg/mL, respectively, with no significant change at 10 µg/mL (Figure 4B). MMP-3 levels showed a modest 11.5% reduction at 2 µg/mL (Figure 4C). MMP-9 secretion decreased by 36.0%, 48.0%, 48.0%, and 44.0% at 0.5, 2, 5, and 10 µg/mL, respectively, relative to the control (Figure 4D). These results indicated that the postbiotic extract promoted ECM preservation by simultaneously enhancing collagen synthesis and reducing MMP-mediated collagen degradation.

### 2.6. Acceleration of Wound Healing by the Postbiotic Extract In Vivo

#### 2.6.1. Wound-Healing Assessment of the Postbiotic Extract In Vivo

**Figure 5:**
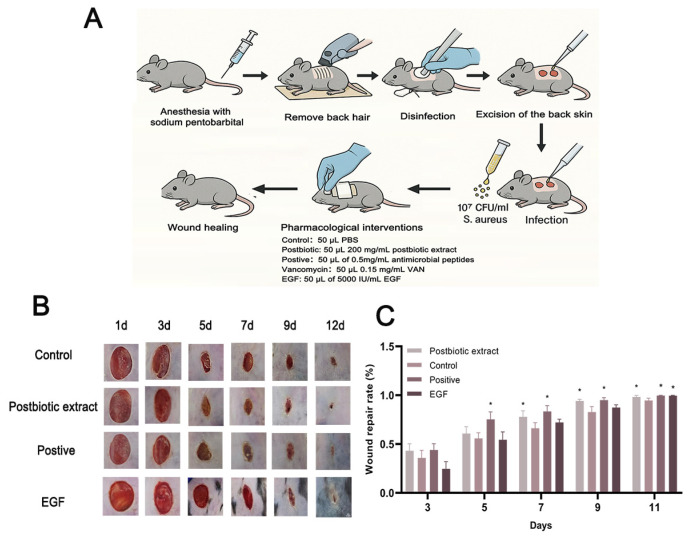
In vivo wound healing assessment with the postbiotic extract. (**A**) Schematic diagram of the mouse wound model and treatment groups. Full-thickness dorsal wounds were created, inoculated with *S. aureus* (10^7^ CFU/mL), and treated twice daily with PBS, postbiotic extract, antimicrobial peptide hLFT-309, or EGF. (**B**) Representative photographs of *S. aureus*-infected wounds treated with PBS (control), postbiotic extract, EGF, and hLFT-309 (positive control). Images were taken at multiple time points post-infection (scale bar = 5 mm). (**C**) Temporal analysis of wound closure. The percentage of wound repair area was measured for each group on days 3, 5, 7, 9, and 11 post-wounding. * *p* < 0.05, vs. control. N = 10 per group.

To further confirm the findings from the in vitro experiments, a full-thickness wound model infected with *S. aureus* was established in mice (Figure 5A). The control group received 50 μL of PBS, while the treatment group was given 50 μL of the postbiotic extract (200 mg/mL). Two positive controls were included: 5000 IU/mL of epidermal growth factor (EGF) and 0.5 mg/mL of antimicrobial peptides (hLFT-309). The objective was to directly compare the effects of the postbiotic extract and antimicrobial peptides (hlf-309), using the biological effects of EGF on cell proliferation, differentiation, and migration as a reference standard. Figure 5B showed significant improvements in wound area reduction, wound edge contraction, and granulation tissue formation in the postbiotic extract group. The fastest wound healing rate was observed on days 7 and 9 of treatment. By day 9, the postbiotic extract group achieved a wound closure rate of 94%, which was 12% higher than the PBS control group and 7% higher than the EGF group, approaching the 96% closure rate of the positive control group—hLFT-309 (Figure 5B,C).

#### 2.6.2. Postbiotic Extract Attenuates Wound Inflammation via Inhibition of Bacterial Growth

**Figure 6:**
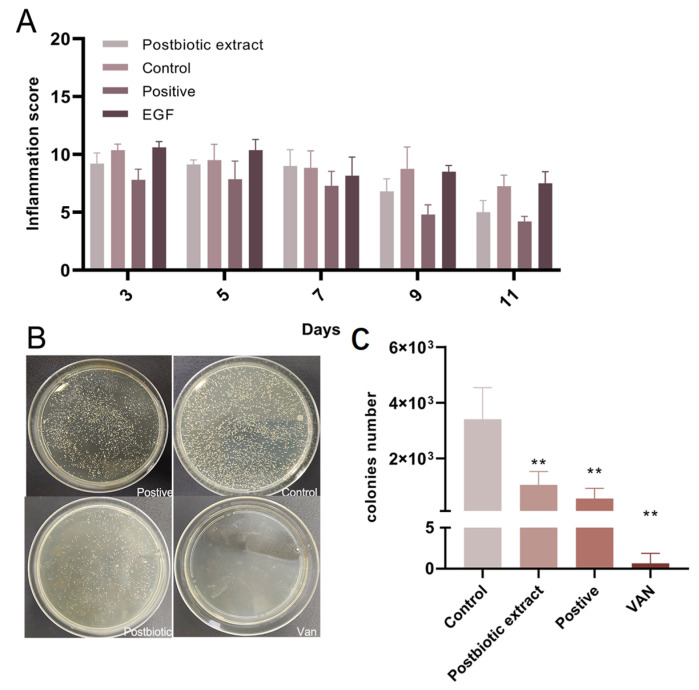
In vivo effects of the postbiotic extract on wound inflammation and bacterial growth. (**A**) Inflammation scores of *S. aureus*-infected wounds following different treatments (PBS control, postbiotic extract, EGF, and positive control: hlf-309). (**B**) Representative images of bacterial cultures obtained from wound secretions on day 3 post-infection, following treatment with PBS, postbiotic extract, positive control (hlf-309) or vancomycin (VAN). (**C**) Quantitative analysis of bacterial colonies grown on tryptic soy broth (TSB) agar plates, measured using Image-Pro Plus 6.0 software (version 6.0, Media Cybernetics, Rockville, MD, USA). ** *p* < 0.01 vs. control. N = 5 per group.

From the day of injury, inflammation in mouse wounds was evaluated every other day using a standardized scoring system. Treatment with the postbiotic extract markedly reduced exudate and prevented purulent and hemorrhagic discharge (Figure 6A). In addition, the postbiotic extract effectively suppressed bacterial proliferation within the wound bed. Antimicrobial efficacy was assessed over three days using the full-thickness wound model, with average bacterial colony counts in the wound beds being 1037 in the control group, 556 in the postbiotic extract group, and about 1 in the vancomycin (VAN) group (Figure 6B,C). These findings were consistent with our in vitro results, further supporting the multifaceted role of the postbiotic extract in promoting wound healing.

#### 2.6.3. The Postbiotic Extract Enhanced Collagen Synthesis in Skin Wound

**Figure 7:**
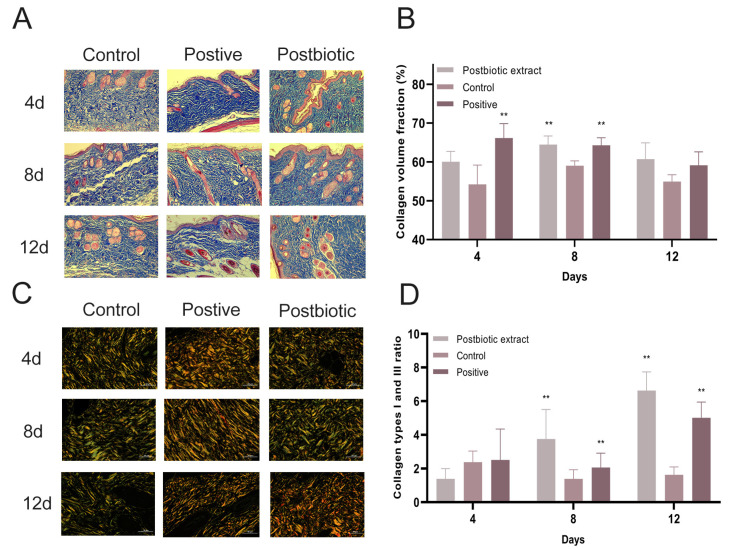
In vivo effects of the postbiotic extract on collagen synthesis and maturation. (**A**) Masson’s trichrome staining of *S. aureus*-infected wound sections collected on days 4, 8, and 12 post-treatment, comparing control, postbiotic extract, and positive control groups. (Scale bar = 100 μm). (**B**) Quantitative analysis of CVF at days 4, 8, and 12 post-treatment in each group. ** *p* < 0.01 vs. control. N = 10 per group. (**C**) Sirius red staining under polarized light illustrating collagen fiber organization. Representative images of wound tissue sections collected on days 4, 8, and 12 show type I collagen (yellow) and type III collagen (green). (Scale bar = 50 μm). (**D**) Temporal changes in the collagen type I/III ratio. Quantification of I-COL/III-COL ratios was performed for each group on days 4, 8, and 12. ** *p* < 0.01 vs. control. N = 10 per group.

Histological examination of mouse wound tissue revealed distinct differences among treatment groups (Figure 7A). Masson’s trichrome staining showed that the postbiotic extract group exhibited enhanced collagen deposition, more uniform collagen distribution, and marked regeneration of skin appendages, whereas the control group displayed sparse collagen fibers, irregular fiber arrangement, and minimal regeneration of skin appendages (Figure 7A). Collagen volume fraction (CVF), defined as the proportion of collagenous area relative to the total dermal tissue area. Quantitative analysis demonstrated a significant increase in collagen volume fraction (CVF) in the postbiotic extract group, resulting in an 11% increase on day 9 compared with the control group and reaching levels similar to those in the positive control group (Figure 7B). A higher ratio of I-COL to Type III collagen (III-COL) is generally indicative of improved healing quality [40,41]. Consistently, a marked reduction in type III collagen and an increase in type I collagen were observed in the postbiotic extract group, leading to a significantly higher I-COL/III-COL ratio compared with the control group (Figure 7C,D).

## 3. Discussion

This study systematically investigated the molecular and cellular mechanisms underlying the wound healing-promoting effect of a postbiotic extract derived from *L. bulgaricus* fermentation. The postbiotic extract, obtained through controlled fermentation followed by inactivation and purification, demonstrated comprehensive therapeutic potential through antimicrobial, immunomodulatory, and tissue regenerative mechanisms. Peptide profiling identified eleven antimicrobial peptides within the extract, classified as bacteriocins, which possess structural features critical for selective targeting of bacterial membranes, including amphipathic domains and specific recognition motifs [42,43,44,45]. In addition, organic acids—mainly lactic and acetic acids—acted synergistically to enhance antimicrobial activity by acidifying the local microenvironment, inhibiting bacterial growth, and increasing membrane permeability [20]. This multi-targeted approach was particularly effective against biofilm-forming pathogens, including *S. aureus and P. aeruginosa* [46], which are resistant to conventional treatments through their protective biofilm barriers [47,48].

The antimicrobial activity of the postbiotic extract was evaluated by determining its MIC against *E. coli*, *S. aureus*, and *P. aeruginosa*. The observed MIC values (6.4–51.2 mg/mL) were relatively high compared with those of conventional antimicrobial agents but were consistent with values reported for other *Lactobacillus*-derived postbiotics, which typically show activity in the mg/mL range [49,50]. Such variability may be influenced by several factors, including the bacterial strain tested, the production and purification methods, and the specific bioactive metabolites present in the extract. Different bacterial strains exhibit distinct resistance profiles, with *P. aeruginosa*, for example, showing intrinsic resistance due to biofilm formation and efflux pumps [51]. Furthermore, differences in the composition of the postbiotic extract, particularly in the abundance of antimicrobial peptides and organic acids, can alter its potency [52]. Fermentation conditions and extraction processes may also impact the concentration and bioactivity of these compounds, contributing to differences in MIC values [53]. Importantly, the therapeutic potential of the extract extends beyond direct antibacterial effects, as its immunomodulatory and regenerative properties also contribute to wound healing—effects that cannot be fully reflected by MIC data alone.Our in vitro and in vivo findings collectively demonstrated that the postbiotic extract exerted a multifaceted effect on wound healing. Its antimicrobial activity was particularly important during the early phase, where it reduced bacterial burden and established a microenvironment favorable for tissue repair. This was accompanied by a significant reduction in TNF-*α* and IL-6 secretion in LPS-stimulated RAW264.7 macrophages, indicating a pronounced immunomodulatory effect. These observations are consistent with previous reports showing that peptidoglycans and extracellular polysaccharides from lactic acid bacteria competitively inhibit TLR2/4 activation and suppress downstream MAPK/NF-κB signaling, thereby reducing pro-inflammatory cytokine production [54,55,56,57,58,59,60,61,62]. Compared with the two positive controls—EGF, which primarily facilitated wound closure during the later stages of healing, and antimicrobial peptide hLFT-309, which combined antimicrobial effects with support for wound contraction and remodeling—the postbiotic extract demonstrated a broader therapeutic profile. Specifically, it controlled bacterial proliferation, reduced excessive inflammatory responses, and promoted collagen remodeling, thereby addressing both infection management and tissue repair in an integrated manner.

In the initial healing phase, keratinocytes play a crucial role through coordinated migration and proliferation. These cells migrate toward the wound center using “leapfrogging” or “rolling” mechanisms, forming a continuous epithelial sheet while proliferating in the basal layer to re-establish the epidermis [63,64,65]. Chronic wounds often exhibit impaired keratinocyte function, characterized by reduced migration, abnormal differentiation, and dysregulated cytokine expression [63]. Our in vitro studies demonstrated that the postbiotic extract ameliorated these pathological conditions by suppressing pathogen growth to create a favorable microenvironment; moderating inflammation to prevent cellular dysfunction; enhancing keratinocyte migration and differentiation. Importantly, the extract also influenced ECM remodeling by significantly increasing type I colla-gen (I-COL) secretion and reducing MMP-1 and MMP-9 secretion, indicating a shift toward matrix preservation. Because excessive MMP activity drives collagen degradation and delays wound closure [66], these effects suggest that the extract helps maintain newly synthesized matrix and supports timely progression to the remodeling phase. Consistent with these in vitro observations, in vivo analysis demonstrated a favorable shift in the collagen I/III ratio following topical application of the postbiotic extract. During normal wound healing, III-COL predominates in early stages, forming a provisional matrix, whereas I-COL provides structural integrity in mature tissue. [67,68]. The gradual transition from III-COL to I-COL marks the progression from proliferative to maturation phase [40,69]. Treatment with the postbiotic extract was associated with increased I-COL deposition, a higher I-COL/III-COL ratio, and more organized collagen fiber architecture, all of which support improved mechanical strength and tissue repair. These beneficial effects on collagen homeostasis are likely mediated by the extracellular polysaccharides (EPS) present in the extract [47]. EPS have been shown to possess strong antioxidant properties [70], which enable the scavenging of free radicals such as DPPH, ABTS, and hydroxyl radicals. By reducing lipid peroxida-tion and reactive oxygen species (ROS) generation—key drivers of collagen degradation—EPS may protect newly syn-thesized matrix components and promote a microenvironment conducive to optimal remodeling [71,72].

The postbiotic extract promoted matrix preservation and stable tissue repair by enhancing type I collagen secretion and suppressing MMP-1 and MMP-9. These effects suggest a regulatory role in ECM remodeling. Previous studies have demonstrated that collagen metabolism and wound healing are tightly controlled by multiple signaling pathways, including Wnt/*β*-catenin and TGF-*β*/Smad cascades, which coordinate fibroblast activity, keratinocyte migration, and ECM turnover [73,74,75,76]. It is therefore plausible that the beneficial effects of the postbiotic extract are mediated through these or related pathways. Further work will be needed to delineate the specific molecular mechanisms involved, which may provide valuable insight for the development of targeted postbiotic-based therapies.

## 4. Materials and Methods

### 4.1. Materials

#### 4.1.1. Cell Lines, Bacterial Strains, and Animals

Human keratinocyte (HaCaT) and mouse mononuclear macrophage (RAW 264.7) cell lines were purchased from Shanghai Zhong Qiao Xin Zhou Biotechnology Co., Ltd. (Shanghai, China). and Procell Life Science & Technology Co., Ltd. (Wuhan, China)., respectively. Cells were cultured in DMEM medium (Gibco, Thermo Fisher Scientific, Waltham, MA, USA) supplemented with 10% fetal bovine serum, penicillin (100 U/mL), and streptomycin (100 U/mL).

*Lactobacillus bulgaricus* (*L. bulgaricus*, CICC 20247), *Escherichia coli* (*E. coli*, CICC 23657), *Staphylococcus aureus* (*S. aureus*, CICC 10384), and *Pseudomonas aeruginosa* (*P. aeruginosa*, CICC 21630) strains were obtained from the China Center of Industrial Culture Collection (CICC). LB broth (Sangon Biotech Co., Ltd., Shanghai, China), tryptic soy broth (TSB), and reinforced clostridium medium (RCM) (Shanghai Acmec Biochemical Co., Ltd., Shanghai, China) were used for bacterial culture.

Eight-week-old specific-pathogen-free (SPF) male C57BL/6 mice were purchased from Liaoning Changsheng Biotech Co., Ltd. (Benxi, China). All animal experiments were approved by the Institutional Animal Care and Use Committee of Dalian Medical University.

#### 4.1.2. Reagents and Assays

Phosphate-buffered saline (PBS), lipopolysaccharide (LPS, from *E. coli* O55:B5; MedChemExpress, Monmouth Junction, NJ, USA), epidermal growth factor (EGF, Kanghesu Biotechnology Co., Ltd., Nanjing, China), antimicrobial peptide hLFT-309 (lab-extracted), and vancomycin (Bioseth Biotechnology Co., Ltd., Zhenjiang, China) were used for treatments. Cell proliferation was assessed using a Cell Counting Kit-8 (CCK-8, Seven Biotech Co., Ltd., Beijing, China).

ELISA kits for interleukin-6 (IL-6, RX203049M) and tumor necrosis factor-alpha (TNF-*α*, RX202412M) were purchased from Ruixinbio Biotechnology Co., Ltd. (Quanzhou, China); I-COL (MM-2023H1) from Jiangsu Meimian Industrial Co., Ltd. (Yancheng, China); and MMP-1 (JL10180), MMP-3 (JL10218), and MMP-9 (JL29650) from Shanghai Jonlnbio Industrial Co., Ltd. (Shanghai, China).

#### 4.1.3. Histological Analysis

Tissue fixation was performed with 4% paraformaldehyde (Servicebio Technology Co., Ltd., Wuhan, China). Masson’s trichrome staining and Sirius Red staining were carried out according to standard protocols. Images were acquired using optical and polarized light microscopy (Leica, Microsystems GmbH, Wetzlar, Germany). All stains and antibodies were supplied by Servicebio.

#### 4.1.4. Equipment

Major instruments used included a cell culture incubator (SANYO Electric Co., Ltd., Osaka, Japan), biosafety cabinet (NuAire Inc., Plymouth, MN, USA), centrifuges (Beckman Coulter Inc., Brea, CA, USA), optical and inverted microscopes (Leica Microsystems GmbH, Wetzlar, Germany), microplate reader (Molecular Devices LLC, San Jose, CA, USA), tissue microtome (Shanghai Leica Instrument Co., Ltd., Shanghai, China), fluorescence imaging system (Wuhan Zhongtai Electronics Co., Ltd., Wuhan, China), illumination system (NIKON, Tokyo, Japan), automatic cell counter (Countess II, Thermo Fisher Scientific Inc., Waltham, MA, USA), TripleTOF 7600 mass spectrometer (AB SCIEX LLC, Framingham, MA, USA), HPLC system (Waters Corporation, Milford, MA, USA), chemiluminescence imaging system (ChemiDoc, Bio-Rad Laboratories Inc., Hercules, CA, USA).

### 4.2. Methods

#### 4.2.1. The Production, Compositional Analysis, and Identification of the Postbiotic Extract

##### The Preparation of the Postbiotic Extract

An aqueous solution of skim milk powder containing 8% milk protein was prepared as the fermentation medium, thoroughly mixed, and sterilized at 110 °C for 15 min. *Lactobacillus bulgaricus* was preserved at −80 °C using glycerol stocks. Before the experiment, 200 µL of the bacterial suspension was inoculated into 10 mL of sterile skim milk medium and incubated aerobically at 37 °C for 8 h to revive the strain. This revival step was repeated 2–3 times to ensure cell viability. The revitalized culture was then inoculated into the fermentation medium at a 2% (*v*/*v*) inoculation rate, achieving a final bacterial concentration of 5.0 × 10^5^ CFU/mL. Fermentation was carried out under aerobic conditions at 37 °C with agitation at 200 rpm for 22 h, after which the culture was heat-inactivated at 121 °C for 20 min.

The fermentation broth was centrifuged at 8000 rpm for 20 min at 4 °C to obtain the supernatant. The supernatant was first filtered through a 10 kDa ultrafiltration membrane, and the resulting permeate was further processed by nanofiltration using a 150 Da molecular weight cut-off membrane. The retentate was collected, freeze-dried to obtain the fermentation powder, and stored at −20 °C until use.

##### The Compositional Analysis of the Postbiotic Extract

The protein content was determined using the Kjeldahl method [77]. Total sugar content was measured by the Phenol-Sulfuric Acid method [78]. Lactose content was analyzed using the Lane-Eynon method [79].

##### The Component Identification of the Postbiotic Extract

Sample preparation: Two milligrams of the postbiotic extract were weighed and completely dissolved in 1 mL of water. The solution was ultrafiltered using a 10 kDa molecular weight cut-off ultrafiltration tube at 14,000× *g* and 20 °C for 20 min. The resulting ultrafiltrate was then lyophilized and stored for further analysis.

Triple TOF™ 7600+ MS analysis: Sample desalting was performed using a C18 column (Waters Oasis HLB SPE column) following previously reported protocols with slight modifications. HPLC-MS/MS analysis was subsequently carried out under the modified conditions [80].

Data processing: The *.wiff files generated by SCIEX OS 3.0 were analyzed using MaxQuant software (version 2.0.3.0, Max Planck Institute of Biochemistry, Martinsried, Germany) against the bovine milk protein database (http://www.uniprot.org/ accessed on 9 July 2023), which contains 69 proteins. Search parameters were set as follows: no enzyme cleavage, no limit for missed cleavages, and no fixed modifications. Methionine oxidation (+15.9949 Da) was specified as a variable modification. The parent ion mass tolerance was set to 50 ppm.

#### 4.2.2. Antimicrobial Activity Assay

The antimicrobial activity of the postbiotic extract was assessed by determining its minimum inhibitory concentration (MIC) and half-maximal inhibitory concentration (IC_50_) against *E. coli*, *S. aureus*, and *P. aeruginosa*. Prior to testing, 200 μL aliquots of each bacterial strain were inoculated into 10 mL of the corresponding liquid medium at a 2% (*v*/*v*) inoculation ratio. The cultures were incubated at 37 °C for 12 h, and this revival process was repeated 2–3 times to ensure full strain reactivation. The bacterial concentration was adjusted by measuring the optical density at 600 nm (OD_600_) using a UV spectrophotometer, followed by dilution to a final concentration of 5 × 10^5^ CFU/mL.

For MIC determination, 100 μL of bacterial suspension was mixed with 100 μL of the postbiotic extract solution at serially diluted concentrations (52.1, 25.6, 12.8, 6.4, 3.2, 1.6, 0.8, 0.4, 0.2, and 0.1 mg/mL) in a 96-well plate. The plates were incubated at 37 °C for 18 h, after which bacterial growth was quantified by measuring OD_600_ using a full-wavelength microplate reader. MIC was defined as the lowest concentration completely inhibiting visible bacterial growth, and IC_50_ values were calculated from the dose–response curves [81].

#### 4.2.3. Inhibition of Lipopolysaccharide (LPS)-Induced Inflammation In Vitro

RAW 264.7 cells were seeded in 96-well plates at 5.0 × 10^4^ cells/well and incubated overnight to allow adherence. To induce inflammation, LPS was added to the model control and treatment wells at a final concentration of 1 μg/mL. After 24 h of LPS stimulation, the postbiotic extract was added to the treatment wells at the indicated final concentrations (0.5, 2, 5, 10 μg/mL), and cells were incubated for an additional 6 h. A non-LPS group (vehicle only) served as the control. At the end of incubation, culture supernatants were collected and centrifuged at 12,000 rpm for 30 min at 4 °C. Clarified supernatants were stored at −80 °C until analysis. IL-6 and TNF-*α* concentrations were determined using commercial ELISA kits according to the manufacturers’ instructions. Briefly, 100 µL of standards or samples were added to antibody-coated wells and incubated at 37 °C for 1 h, followed by washing and incubation with HRP-conjugated detection antibodies. After color development with TMB substrate, the reaction was stopped with 50 µL of stop solution, and absorbance was measured at 450 nm using a microplate reader. Cytokine concentrations were calculated from standard curves. Successful model induction was verified by the elevated cytokine levels in the LPS-only group compared with the non-LPS control.

#### 4.2.4. Cell Proliferation Assay

HaCaT cells were used for cell proliferation analysis using the CCK-8 assay. Cells were seeded into 96-well plates at a density of 1.5 × 10^4^ cells/well and allowed to adhere for 12 h. The postbiotic extract was then added to each well to achieve the desired final concentrations (0.5, 2, 5, 10 μg/mL), and cells were incubated for 24 h. Subsequently, 10 μL of CCK-8 solution was added to each well, followed by incubation for an additional 1–4 h. Absorbance was measured at 450 nm using a microplate reader.

The cell proliferation rate was calculated using the following formula:

$$Cell\;proliferation\;rate=\frac{A_{control}-A_{0}}{A_{x}-A_{0}}\times 100\%$$

where *A*_0_ represents the blank well, *A_x_* reflects the absorbance of cells treated with the postbiotic extract, and *A*_control_ represents the absorbance of cells treated with none of the postbiotic extract. Cell proliferation rates were calculated using absorbance data from the CCK-8 assay and analyzed with GraphPad Prism software (version 9.5.0, GraphPad Software, LLC, San Diego, CA, USA) [82].

#### 4.2.5. Keratinocyte Migration Assay

HaCaT keratinocytes were seeded into 2-well Culture-Inserts placed in 6-well plates at a density sufficient to reach confluence within 24 h. After a confluent monolayer had formed, the inserts were carefully removed with sterile forceps to create cell-free gaps. The wells were washed twice with PBS to remove detached cells and then incubated in serum-reduced medium containing the postbiotic extract (0.5–10 µg/mL) or vehicle control. Images were taken at 0 h (immediately after insert removal), 8 h, and 24 h using an inverted microscope. Gap areas were quantified using ImageJ software (version 1.53t, National Institutes of Health, Bethesda, MD, USA), and cell migration was expressed as the percentage of wound closure relative to 0 h.

#### 4.2.6. Measurement of I-COL MMP-1, MMP-3, and MMP-9 (ELISA)

HaCaT cells were seeded into 96-well plates at a density of 5.0 × 10^4^ cells/well and cultured overnight to allow adherence. After cells attached, the postbiotic extract was added to the treatment wells at various final concentrations, while the control wells received PBS alone. Following 24 h of incubation, the culture supernatants were collected and centrifuged at 12,000 rpm for 30 min at 4 °C to remove cell debris. The clarified supernatants were stored at −80 °C until analysis.

I-COL, MMP-1, MMP-3, and MMP-9 concentrations were quantified using ELISA kits according to the manufacturers’ protocols. Briefly, 100 µL of standards or samples was added to antibody-coated wells and incubated at 37 °C for 1 h. Plates were washed three times with 1× wash buffer, followed by the addition of 100 µL of HRP-conjugate working solution and incubation at 37 °C for 30 min. After washing five times, 90 µL of TMB substrate was added and incubated for 15 min at 37 °C in the dark. The reaction was stopped by adding 50 µL of stop solution, and absorbance was measured at 450 nm (OD_450_) using a microplate reader. Analyte concentrations were calculated from standard curves generated for each plate and expressed as ng/mL.

#### 4.2.7. Infected Full-Thickness Wound Model

Male C57BL/6 mice were used to establish an infected full-thickness wound model. Mice were anesthetized by intraperitoneal injection of 1% sodium pentobarbital prior to surgery. After shaving the dorsal hair, two symmetrical full-thickness skin wounds (0.8 cm in diameter) were created on the depilated back skin using a sterile biopsy punch. Each wound was inoculated with 50 μL of *S. aureus* suspension at a concentration of 10^7^ CFU/mL [83]. At the end of each experiment, mice were euthanized by CO_2_ inhalation, and death was confirmed by cessation of respiration and heartbeat. This wound model was applied for the subsequent animal experiments described in Section 4.2.8 and Section 4.2.9.

#### 4.2.8. In Vivo Study of Antimicrobial Efficacy

After establishing the wound model, mice were randomly assigned to four groups (10 mice per group, except for the VAN group (n = 5)): control, postbiotic extract, antimicrobial peptide hLFT-309 (positive control), and vancomycin (VAN). Treatments were applied to both dorsal wounds twice daily as follows: 50 μL PBS, 50 μL of 200 mg/mL postbiotic extract, 50 μL of 0.5 mg/mL antimicrobial peptide hLFT-309, or 50 μL of 0.15 mg/mL VAN (PBS used as solvent) [39].

Antimicrobial peptide hLFT-309 is a synthetic antimicrobial peptide derived from human lactoferrin, previously characterized for its antimicrobial and wound-healing activities [39]. The peptide (sequence: GSPSGQKDLLF) was synthesized by Synpeptide Co., Ltd. (Shanghai, China) using solid-phase peptide synthesis, with a purity greater than 95% as determined by HPLC and a molecular weight of 1148.28 Da. Its physicochemical properties, including a hydrophobicity index of −0.409, were characterized in prior studies [84].

On day 3 after surgery, wound secretions were collected from each group, diluted with 5 mL of PBS, and 100 μL of each dilution was spread evenly onto TSB agar plates. The plates were incubated at 37 °C for 18 h, and bacterial colonies were photographed and quantified using Image-Pro Plus 6.0 software (version 6.0, Media Cybernetics, Rockville, MD, USA).

#### 4.2.9. Quantification of In Vivo Wound Healing

Infected full-thickness wound models were established as described previously, and mice were randomly divided into four treatment groups (N = 10 per group): epidermal growth factor (EGF), control, postbiotic extract, and antimicrobial peptide hLFT-309 (positive control). The EGF group received 50 μL of 5000 IU/mL EGF, the control group received 50 μL of PBS, the postbiotic extract group received 50 μL of 200 mg/mL extract, and the antimicrobial peptide group received 50 μL of 0.5 mg/mL peptide (hlF-309).

Treatments were administered twice daily for 12 consecutive days. Wound areas were photographed using a digital camera at predetermined time points. The wound repair rate was calculated from the photographs using ImageJ (National Institutes of Health, Bethesda, MD, USA) by tracing the wound margins and measuring the pixel area. Measurements were performed in triplicate, and the mean values were used to calculate the percentage of closure relative to the original wound area according to the formula:

$$Wound\;repair\;rate=\left[1-\left(\frac{S_{n}}{S_{1}}\right)\right]\times 100\%$$

where *S_n_* and *S*_1_ were, respectively, the area of the wound in mice on day n and day 1.

At the same time, the inflammation score was recorded every two days. Inflammation score modified and simplified from the Bates-Jensen Wound Assessment Tool [85]: (1) Wound edge: 1 = blurred, unable to distinguish the wound edge; 2 = able to clearly distinguish the wound edge; 3 = distinguishable contour, with the base of the wound lower than the wound edge. (2) Exudate type: 1 = no exudate; 2 = thin light red or pink color exudate; 3 = red or pink color exudate. (3) Exudate volume: 1 = no exudate, wound tissue dry; 2 = wound tissue slightly moist, soaking 0-25% of the dressing; 3 = wound tissue moist, soaking more than 25% of the dressing. (4) Wound color: 1 = normal or pink; 2 = light red; 3 = Dark red or purple.

#### 4.2.10. Histological Analysis

At 4-day intervals, full-thickness skin samples (including the wound and a 0.5 cm margin of adjacent normal tissue) were harvested from the dorsal site. The tissues were fixed in 4% paraformaldehyde for histological slide preparation. Slides were subjected to Masson’s trichrome and Sirius red staining. Images of stained samples were captured using an optical microscope and polarized light microscopy. Collagen volume fraction (CVF) and the ratio of collagen type I to type III were analyzed and calculated from Masson’s trichrome and Sirius red staining images, respectively, using the Image-Pro Plus 6.0 software (version 6.0, Media Cybernetics, Rockville, MD, USA).

#### 4.2.11. Statistical Analysis

Statistical analysis was performed by one-way or two-way analysis of variance (ANOVA) with multiple comparisons test or Student’s *t*-test using GraphPad Prism version 9.5 and IBM SPSS Statistics 25 (IBM SPSS, Armonk, NY, USA). *p* < 0.05 was considered statistically significant. Results are shown as mean ± SEM. Graphs were generated by using GraphPad Prism version 9.5.

## 5. Conclusions

This study demonstrates that a composite postbiotic extract derived from *Lactobacillus bulgaricus* promotes wound healing through complementary antimicrobial, immunomodulatory, and pro-regenerative effects. In vitro, the extract significantly enhanced keratinocyte proliferation, stimulated I-COL secretion, and reduced MMP-1 and MMP-9 levels, suggesting a shift toward ECM preservation. In vivo, topical application accelerated wound closure, increased the I-COL/III-COL ratio, and improved collagen organization, indicating enhanced tissue remodeling and maturation. Collectively, these findings support the potential of postbiotic extracts as safe, cell-free therapeutic candidates or adjuncts to conventional treatments, particularly for infected or chronic wounds.

Future studies should elucidate the molecular pathways underlying these effects, including potential regulation via Wnt/*β*-catenin and TGF-*β*/Smad signaling, to better define the mechanisms of ECM remodeling and immune modulation. Long-term safety assessments and formulation optimization for different wound types are also warranted. Furthermore, combinatorial approaches that integrate postbiotics with existing wound care modalities may further enhance their therapeutic efficacy.

## Figures and Tables

**Table 1:** MIC and IC_50_ of the postbiotic extract against *S. aureus*, *E. coli. and P. aeruginosa.*

	MIC (mg/mL)	IC_50_ (mg/mL)
*S. aureus*	51.2	26.3
*E. coli*	6.4	1.7
*P. aeruginosa*	51.2	28.9

**Table 2:** Composition of the postbiotic extract (g/100 g).

	Protein	Total Soluble Sugars	Lactose
Substrate	35.74 ± 0.29	38.61 ± 2.13	32.21 ± 0.55
Postbiotic extract	9.85 ± 0.49	50.73 ± 1.29	46.88 ± 1.85

**Table 3:** The antimicrobial peptides in the postbiotic extract.

Protein Precursor	Peptide Sequence	References
Alpha-S1-casein	VLNENLLR	[30]
Alpha-S2-casein	TKVIPYVRYL	[31]
Beta-casein	TEDELQDKIHPF	[32]
	DVENLHLPLPL	[33]
	AVPYPQR	[34,35]
	YQEPVLGPVRGPFPI	[32,36]
	YQEPVLGPVRGPFPIIV	[32]
Kappa-casein	YYQQKPVA	[37]
	MAIPPKKNQDKTEIPTINT	[38]
	VESTVATL	[37]
	NLENTVKETIKYLKSLFSHAFEVVKT	[39]

## Data Availability

The original contributions presented in this study are included in the article. Further inquiries can be directed to the corresponding authors.
